# Cross-sectional study of household solid fuel use and renal function in older adults in China

**DOI:** 10.1016/j.envres.2022.115117

**Published:** 2022-12-20

**Authors:** Thirumagal Kanagasabai, Ellison Carter, Li Yan, Queenie Chan, Paul Elliott, Majid Ezzati, Frank Kelly, Gaoqiang Xie, Xudong Yang, Liancheng Zhao, Dongshuang Guo, Stella S. Daskalopoulou, Yangfeng Wu, Jill Baumgartner

**Affiliations:** 1 School of Population and Global Health, McGill University, Montreal, Quebec, Canada; 2 Department of Civil and Environmental Engineering, Colorado State University, Fort Collins, Colorado, USA; 3 Department of Epidemiology and Biostatistics, and MRC Centre for Environment and Health, School of Public Health, Imperial College London, London, UK; 4 Environmental Research Group, MRC Centre for Environment and Health, School of Public Health, Imperial College London, London, UK5Peking University Clinical Research Institute; 5 Peking University Health Science Center, Beijing, China; 6 Department of Building Science, Tsinghua University, Beijing, China; 7 Fu Wai Hospital and Cardiovascular Institute, Chinese Academy of Medical Sciences, Beijing, China; 8 Department of Cardiology, Yuxian Hospital, Yuxian, Shanxi, China; 9 Department of Medicine, Division of Internal Medicine and Division of Experimental Medicine, McGill University, Montreal, Quebec, Canada

**Keywords:** household air pollution, estimated glomerular filtration rate, chronic kidney disease

## Abstract

**Background:**

Emerging evidence links outdoor air pollution and declined renal function but the relationship between household air pollution and renal function is poorly understood.

**Methods:**

Using cross-sectional data from the multi-provincial INTERMAP-China Prospective Study, we collected blood samples and questionnaire information on stove use and socio-demographic factors. We calculated estimated glomerular filtration rate (eGFR) from serum creatinine to assess renal function. Participants with eGFR <60 mL/min per 1.73 m2 were defined as having chronic kidney disease (CKD) in this analysis. Generalized estimating equations were used to estimate the association of household fuel use with renal function and prevalent CKD in models adjusting for confounders.

**Results:**

Among the 646 enrolled adults (40-79y; 56% female), one-third exclusively used clean fuel (gas and electric) cookstoves and 11% of northern China participants (n=49 of 434) used only clean fuel heaters, whereas the rest used solid fuel. In multivariable models, use of solid fuel cookstoves was associated with 0.17 ml/min/1.73 m2 (95% CI: - 0.30, 0.64) higher eGFR and 19% (0.86, 1.64) higher prevalence of CKD than exclusive clean fuel use. Greater intensity of solid fuel use was associated with 0.25 ml/min/1.73 m2 (-0.71, 0.21) lower eGFR per 5 stove-use years, though the confidence intervals included the null, while greater current intensity of indoor solid fuel use was associated with 1.02 (1.00, 1.04) higher prevalent CKD per 100 stove-use days per year. Larger associations between current solid fuel use and intensity of use with lower eGFR and prevalent CKD were observed among participants in southern China, those with hypertension or diabetes (eGFR only), and females (CKD only), through these groups had small sample sizes and some confidence intervals included the null.

**Conclusion:**

We found inconsistent evidence associating household solid fuel use and renal function in this cross-sectional study of peri-urban Chinese adults.

## Introduction

Chronic kidney disease (CKD) is an important contributor to morbidity and mortality from non-communicable diseases, affecting over 10% of the global population (Hill et al., 2016) and responsible for an estimated 1.2 million premature deaths in 2017 (Bikbov et al., 2020). CKD-related mortality is expected to increase to 2.2 million by 2040 in the best-case scenario and up to 4.0 million in the worst-case scenario, the majority of which will occur in low- and middle-income countries like China as their population ages and urbanizes (Bikbov et al., 2020; Zhang et al., 2012). Kidney failure, which is the most advanced stage of CKD, requires costly dialysis or kidney transplant and causes considerable medical, economic and social burden to patients, their families, and health care systems (Jha et al., 2013; Webster et al., 2017). Individuals with CKD are also at increased risk of developing cardiovascular diseases (CVD) and dying from it, which are the leading contributors to the global disease burden (Jankowski et al., 2021).

Chronic kidney disease and CVD share many known risk factors including smoking, unhealthy diet, insufficient physical activity, hypertension, and diabetes (Weiner et al., 2006), and possibly also exposure to environmental risks like air pollution (Ye et al., 2021). Exposure to outdoor air pollution is a well-established cardiovascular risk factor (Lelieveld and Münzel, 2020; Zhang et al., 2011), with one hypothesized mechanism being that inhaled particles can induce systemic inflammation and oxidative stress (Chin, 2015), which could also damage the kidneys and affect renal function (Swaminathan and Shah, 2011). Studies of urban populations in North America and Asia found associations of outdoor fine particulate matter (PM_2.5_) on prevalent and incident CKD, and progression to kidney failure (Lin et al., 2020; Liu et al., 2020; Wu et al., 2020; Ye et al., 2021), though studies of outdoor PM_2.5_ and biomarkers of renal function were inconsistent (Feng et al., 2021), possibility due to differences in the biomarkers used to assess kidney function, the diversity in exposure assessment methods, and inconsistent consideration of confounders.

Exposure to air pollution can also arise from household combustion of solid fuel (i.e., coal and biomass), which emits fine particulate matter and other air pollutants into homes and communities (Lai et al., 2019) to levels that can far exceed urban air pollution (Health Effects Institute, 2020; Luo et al., 2021). An estimated 3.8 billion people globally and 450 million people in China primarily cook with solid fuel-burning stoves. Exposure to household air pollution from burning solid fuel is increasingly recognized as a CVD risk factor, though less understood is whether exposure to household air pollution also impacts kidney function. In the two previous studies of household air pollution and kidney function in China, primary users of indoor solid fuel stoves had lower kidney function than primary users of electric or gas stoves (Xue et al., 2021) and females with a history of coal cookstove use had higher kidney disease-related mortality (Kim et al., 2016), though neither study captured supplemental stove uses, long-term fuel use trends, or intensity of solid fuel use, which could have different effects than primary fuel use (Jewitt et al., 2020). Other studies of solid fuel users in China, India, and Honduras found associations between exposure to particulate matter air pollution and higher blood pressure, which is a risk factor for glomerular filtration rates (GFR) decline and CKD (Baumgartner et al., 2018; Bowe et al., 2020; Norris et al., 2016; Young et al., 2019). These studies motivate further investigations of household air pollution and kidney function that use more detailed exposure assessments and that account for socioeconomic status, comorbidities, and geographic differences that may impact behavioural and environmental exposures (Liu et al., 2020; Wu et al., 2020).

In this context, we investigated the associations of current and long-term solid fuel stove use and intensity of use with kidney function and prevalent CKD in Chinese adults enrolled in the International Study of Macro/Micronutrients and Blood Pressure (INTERMAP)-China Prospective (ICP) Study, a multi-provincial study that included detailed measurement of household fuel use, including current and historical intensities of fuel use practices, blood creatinine-based estimated GFR (eGFR), and a comprehensive set of covariates (Yan et al., 2019).

## Methods

### Study setting

Our study took place in 17 villages located in northern (Pinggu County, Beijing: N40°8′, E117°6′; Yu County, Shanxi: N38°05′, E113°24′) and southern China (Wuming County, Guangxi: N22°49’, E108°18′) that represent lower-income areas with household energy use practices that are characteristic of peri-urban and rural China. More information about the study setting is provided elsewhere (Yan et al., 2019).

### Study design and participants

The ICP Study is a longitudinal study that was established to identify environmental and nutritional risk factors for chronic disease. Details on the study design and participants are described elsewhere (Yan et al., 2019). Briefly, of the 782 participants (40-79 years of age; 55% female) enrolled in the ICP Study between 2015-2016, most (n=574, aged 60-79 years in 2015; 85% response rate) were previously enrolled in the INTERMAP-China study in 1995-97, which randomly sampled households in the study villages and then randomly selected one adult from each household to participate (Stamler et al., 2003; Yan et al., 2019). The remaining 208 ICP study participants (aged 40-59 years in 2015; 88% response rate) were randomly selected from village rosters. The present study uses cross-sectional data collected during the 2015-2016 wave of the ICP study. We obtained written informed consent from participants and the study protocols received ethical approvals from all investigator institutions.

### Data collection

Trained staff conducted the ICP study measurements in Beijing from December 2015 and September 2016, in Shanxi from August 2015 and November 2015, and in Guangxi from November 2016 (Yan et al., 2019). Briefly, we conducted two campaigns in the northern provinces to capture the heating and non-heating seasons, which are known to affect environmental conditions, including outdoor sources of air pollution, outdoor temperature, and other meteorological variables, and behaviours including stove use, time spent indoors and outdoors, and activity level, whereas only one campaign was conducted in the temperate and sub-tropical Guangxi site. In each campaign, we administered structured questionnaires and performed anthropometric measurements (height, weight, waist circumference, and blood pressure). In the second campaign in Beijing and Shanxi and the single campaign in Guangxi, we also collected blood samples. Study staff underwent a training program prior to each campaign, and strict quality control procedures were followed throughout data collection and described elsewhere (Yan et al., 2019).

### Household fuel use and intensity of use

Staff administered an image-based household energy questionnaire used to construct a set of categorical and continuous fuel-use variables that characterized the current and historical use of indoor solid fuel stoves (Carter et al., 2019). Briefly, participants identified all stoves ever used by their household at present and over the past 20 years and reported, for each stove, the type of fuel used, location of use, duration of use in 5-year intervals, and frequency of use. Staff conducted home visits to verify the accuracy of self-reported stove information in a 5% randomly-selected subsample of participant homes. Using methods described elsewhere (Tseng et al., 2022), we used this information to construct the following four stove use metrics: ‘Current stove use’ for (1) cooking and (2) heating at the time of the survey, defined as either exclusive use of clean fuel (i.e., gas or electric) or any use of solid fuel. ‘Intensity of indoor solid fuel use’ derived from information on the duration and frequency of use, and measured by (3) the total number of solid fuel stove-use days in the past year, and (4) the number of solid fuel stove-use years over the past 20 years (Table S1).

### Assessment of kidney function

Clinical staff collected venipuncture blood samples from participants in their village clinics, which were cold chain transported to a local hospital within 2h of collection and analyzed using an automatic biochemical analyzer following standardized procedures (Yan et al., 2019). We calculated the eGFR with serum creatinine using the Chronic Kidney Disease Epidemiology Collaboration (CKD-EPI) equation adapted to the Chinese population (Table S2). The latter has been shown to perform better at predicting renal function decline in Chinese adults than the Modification of Diet in Renal Disease equation adapted to the Chinese population (Chi et al., 2017; Wang et al., 2014). Participants with an eGFR <60 mL/min per 1.73 m^2^ (stage ≥3A), which indicates greater than a mild loss of renal function (Lamb et al., 2013), were defined as having CKD in this analysis. Participants that responded yes to the question “have you ever been told by a physician that you have kidney disease?” were considered as having physician-diagnosed kidney disease.

### Covariates

Staff administered household and individual questionnaires to collect information on household demographics, socioeconomic status, and chronic disease risk factors including age, sex, yearly household income, educational attainment, alcohol consumption, history of tobacco smoke exposure, physical activity, and medical history (see variables and their categories in Table 1). Alcohol consumption was categorized as: never; occasional if consumed <1 drink per week; or regular if consumed ≥1 drink per week. History of tobacco smoking was categorized as: never; former if they had at least 100 cigarettes but were not currently smoking; or current. Secondhand smoke exposure, defined as the presence of a smoker in household for 6 months or more, was categorized as never, former, and current. Height, weight, waist circumference, and blood pressure were measured in each campaign using procedures described elsewhere (Kanagasabai et al., 2022; Yan et al., 2019). Serum concentrations of triglycerides, lipoprotein cholesterols (total cholesterol, HDL cholesterol, and LDL cholesterol), and plasma glucose were analyzed using standard methods (Yan et al., 2019).

Hypertension was defined as systolic blood pressure ≥140 mmHg, and/or diastolic blood pressure ≥90 mmHg, and/or a self-reported history of anti-hypertension medication use. Diabetes was defined as physician-diagnosed diabetes based on self-report by the participant or fasting blood glucose ≥7.0 mmol/L. Participant body mass index (BMI) was calculated as weight in kilograms divided by height in meters squared. The Working Group on Obesity in China recommends BMI cut-offs of ≥24.0 kg/m^2^ to define overweight and ≥28.0 kg/m^2^ to define obesity (Pan et al., 2021). Participants responding yes to the question of “have you ever been diagnosed of coronary heart disease or myocardial infarction in a hospital? or who reported being hospitalized in the past 12 months for coronary heart disease” were classified as having coronary heart disease, and participants responding yes to the question “have you ever been diagnosed of stroke or transient ischemic attack?” or who reported being hospitalized in the last 12 months for stroke or transient ischemic attack were classified as having a previous stroke. Participants were considered as having CVD if they had reported a history of coronary heart disease, myocardial infarction, stroke, or transient ischemic attack.

### Statistical analysis

Generalized estimating equation (GEE) linear models that accounted for province-level clustering were used to estimate the cross-sectional associations between exposure to household solid fuel use and renal function measured by eGFR (Hubbard et al., 2010). The associations between household solid fuel use and prevalent CKD were determined using modified Poisson regression models, which provide consistent estimates and are more stable than the GEE binomial (Pedroza and Truong, 2017), estimating prevalence ratios (PR) and 95% confidence intervals (95% CI).

Multivariable models were adjusted for variables selected *a priori* that could conceivably confound the associations of household solid fuel use and renal function based on a review of the literature (Bowe et al., 2018; Yang Ya-Ru et al., 2017), including age, sex, waist circumference, yearly household income, education attainment, alcohol consumption, physical activity, history of tobacco smoking, secondhand smoke exposure, and systolic blood pressure. Waist circumference and BMI were highly correlated (r=0.8); thus, we adjusted only for waist circumference which was the stronger confounder in the multivariable models.

Missing data on yearly household income (N_clean_=36; N_solid_=33), waist circumference (N_clean_=0; N_solid_=7), and body mass index (N_clean_=1; N_solid_=7) were handled with multiple imputation, under a missing at random (MAR) assumption. Missing variables were imputed using the Multivariate Imputation by Chained Equations (mice) package in R that uses the Markov Chain Monte Carlo (MCMC) method to assess the correlation structure of the data and imputes missing values for each incomplete variable five times by regression of incomplete variables on the other variables iteratively (Zhang, 2016). Variables included in the imputation process included age, sex, physical activity, alcohol consumption, smoking status, secondhand smoke exposure, occupation, and education.

Patterns of missing were assessed for systematic differences with the md.pattern function in mice. We found similar mean eGFR, age, sex, and smoking status for participants with missing household income information versus those with complete data, supporting the MAR assumption (Supplementary Table S3). Following imputation, distributions of the observed and imputed data for variables were compared and found to be similar (Supplementary Table S4).

We observed similar eGFR results and key demographic and behavioral variables for participants with and without complete household income data, the most common missing variable in our study, supporting the imputation assumption that these data were missing at random (Table S3).

Based on findings from previous studies (Bowe et al., 2018, 2020; J. Wang et al., 2017; Yang Ya-Ru et al., 2017), we assessed possible effect size modification (p <0.10) by study region, sex (males versus females), median age (<63y versus ≥63y, for eGFR models), hypertension, and diabetes by creating product terms between fuel use variables and the potential modifiers.

As sensitivity analyses, we conducted our multivariable models limited only to participants with high BMI (≥28 kg/m^2^) (n=159), which increases CKD and kidney failure risks (Stenvinkel et al., 2013), and after excluding participants with a history of coronary heart disease or stroke (n=77) as their renal function may be compromised (Nakayama et al., 2007). We additionally adjusted (1) for heating fuel in models with cooking fuel use as the exposure, and (2) for cardiovascular or metabolic conditions that could be confounders or variables along the causal pathway between household solid fuel use-eGFR/CKD including diabetes and hypertension (Baumgartner et al., 2018; Bowe et al., 2020; Yu et al., 2018). Statistical analyses were conducted in R (www.r-project.org; version 3.5.3) using the *geepack* package. The STROBE checklist for our study is provided in Table S5.

## Results

Our analysis included 646 participants who completed the household fuel use questionnaires (n=753) and provided whole blood samples for biochemistry analysis (n=689), resulting in an analytical sample of 646 participants. The mean participant age was 62.6y (range: 40-79y) at the first visit and 56% were female (Table 1). Most participants (84%) had completed primary school, over half of whom also completed secondary school. Nearly a quarter (23%) of participants were current tobacco smokers; among never smokers, 17% were former smokers and 44% lived with a smoker. Over half (53%) had hypertension, 19% had diabetes, and 12% reported a history of coronary heart disease or stroke.

A third (33%) of participants exclusively used clean fuel cookstoves at the time of the questionnaire. Exclusive users of clean fuel cookstoves were on average younger, had higher household income and lower systolic blood pressure, were less physically active, and were less likely to report having physician-diagnosed kidney disease than solid fuel users (Table 1).

A higher percentage of participants in Shanxi reported current exclusive use of clean fuel cookstoves (40%) and heating stoves (17%) compared with Beijing and Guangxi (Table 2). However, intensity of indoor solid fuel use, as measured by solid fuel stove-use days in the past year and solid fuel stove-use years over the past 20 years, was highest among Shanxi participants.

Participant eGFR ranged from 18.2 to 123.5 ml/min/1.73 m^2^ [mean (SD)=82.4 (15.2)], and 53 participants (8%) were below the eGFR cut-off for CKD of <60 ml/min/1.73 m^2^ (Table 2). Participants in southern China (Guangxi) had, on average, 10 ml/min/1.73 m^2^ lower eGFR than those in northern China and a higher prevalence of eGFR-indicated CKD (Table 2) and self-reported previous diagnosis of kidney disease by a physician (Table S6). Of the 22 participants who reported physician-diagnosed kidney disease, only 4 had eGFR <60 ml/min/1.73 m^2^.

**Figure 1:**
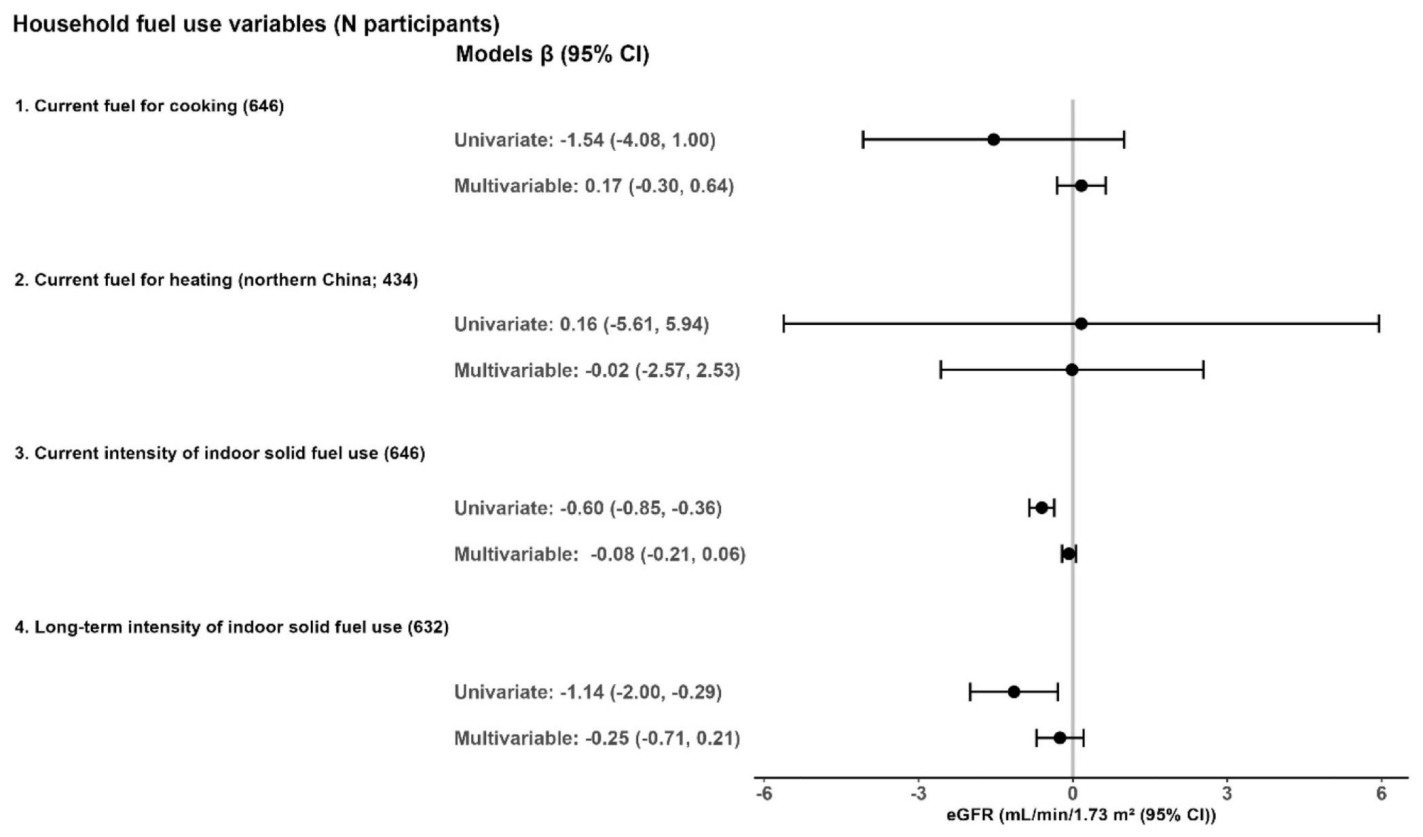
Associations between household solid fuel use and eGFR in older Chinese adults. CI = confidence interval, eGFR = estimated glomerular filtration rate Estimates are for solid fuel exposures compared to reference groups (1 = current exclusive use of clean fuel cookstoves; and 2 = current exclusive use of clean fuel heating stoves). Units of exposures for 3 and 4 are per 100 solid fuel stove-use days per year and per 5 solid fuel stove-use years, respectively. Multivariable models adjusted for age, sex, waist circumference, systolic blood pressure, educational attainment, yearly household income, alcohol consumption, history of tobacco smoking, secondhand smoke exposure, and physical activity. All models accounted for province-level clustering. A directed acyclic graph is provided in Figure S1.

**Figure 2:**
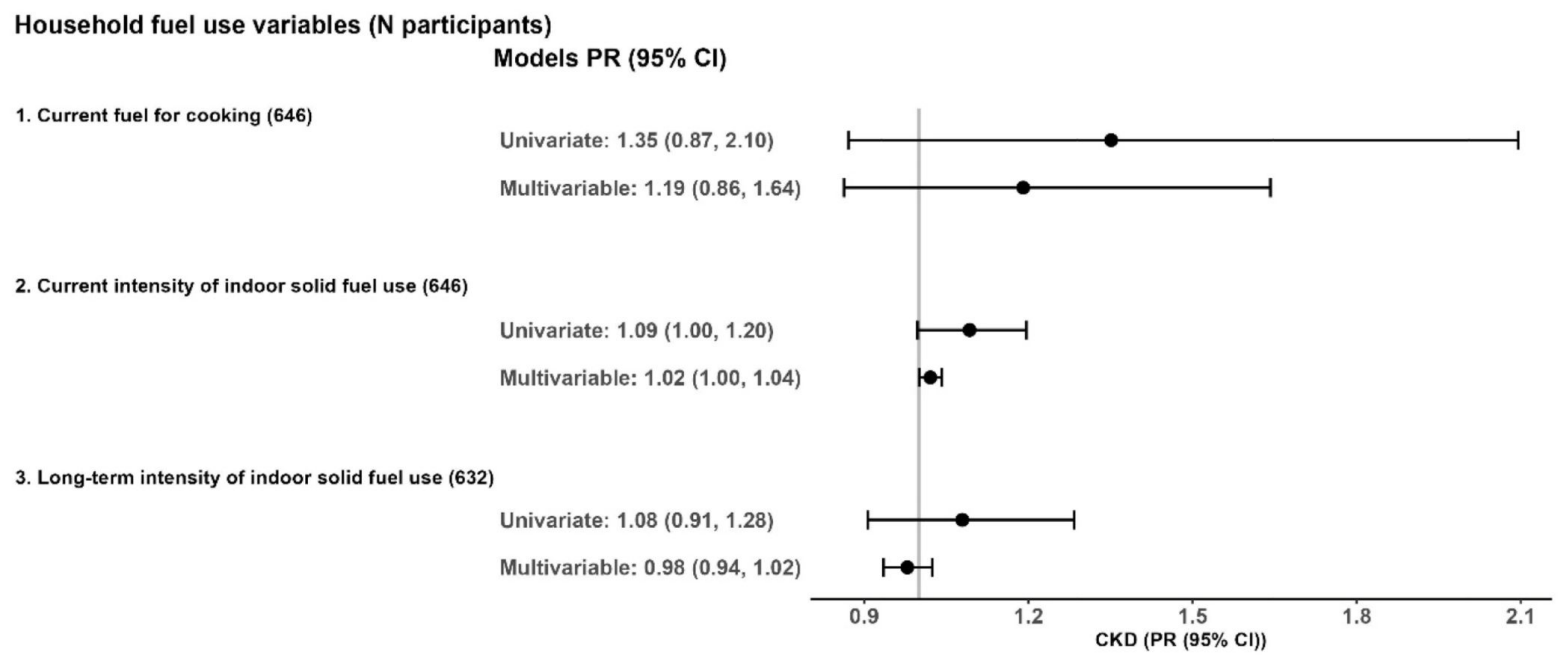
Associations between household solid fuel use and prevalent CKD in peri-urban Chinese adults. CI = confidence interval, CKD = chronic kidney disease, PR = prevalence ratio Estimates are for solid fuel exposures compared with reference groups (1 = current exclusive use of clean fuel cookstoves). Number of participants with CKD by cooking fuel: N_clean_ = 14; N_solid_ = 39. Units of exposures for 2 and 3 are per 100 solid fuel stove-use days per year and per 5 solid fuel stove-use years, respectively. Multivariable model adjusted for age, sex, waist circumference, systolic blood pressure, educational attainment, yearly household income, alcohol consumption, history of tobacco smoking, secondhand smoke exposure, and physical activity. All models accounted for province-level clustering.

In the multivariable models with all participants (Figure 1), we did not find strong or consistent associations between solid fuel stove use or intensity of use with renal function, as measured by eGFR. Current use of solid fuel for cooking and greater intensity of solid fuel use were associated with a slightly higher prevalence of CKD (1.02 (1.00, 1.04)), though confidence intervals included the null (Figure 2).

**Figure 3:**
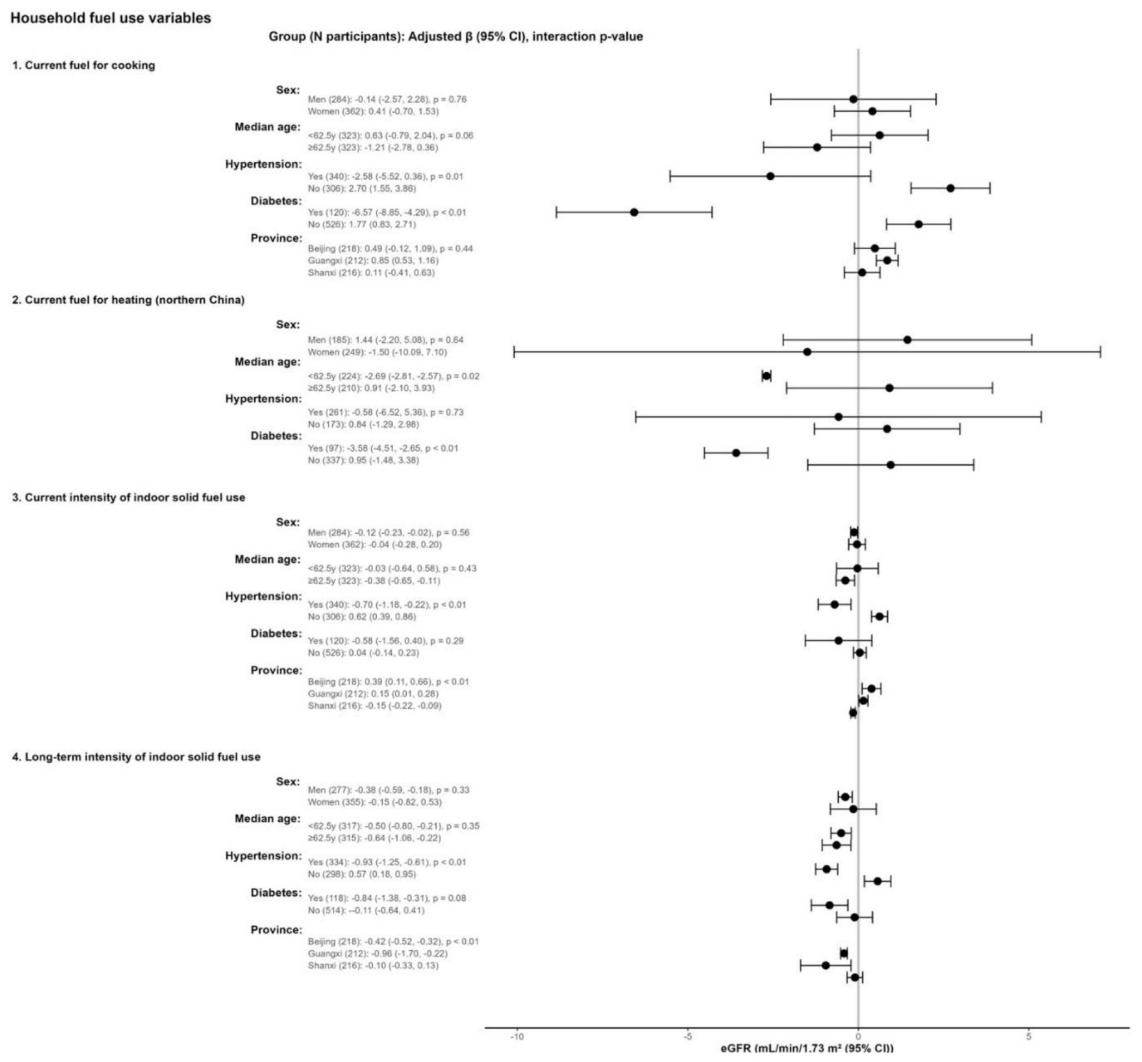
Associations between household solid fuel use and eGFR for different subgroups. CI = confidence interval, eGFR = estimated glomerular filtration rate Estimates are for solid fuel exposures compared to reference groups (1 = current exclusive use of clean fuel cookstoves; and 2 = current exclusive use of clean fuel heating stoves). Units of exposures for 3 and 4 are per 100 solid fuel stove-use days per year and per 5 solid fuel stove-use years, respectively. Multivariable model were adjusted for age, sex, waist circumference, systolic blood pressure, educational attainment, yearly household income, alcohol consumption, history of tobacco smoking, secondhand smoke exposure, and physical activity. The models accounted for province-level clustering.

**Figure 4:**
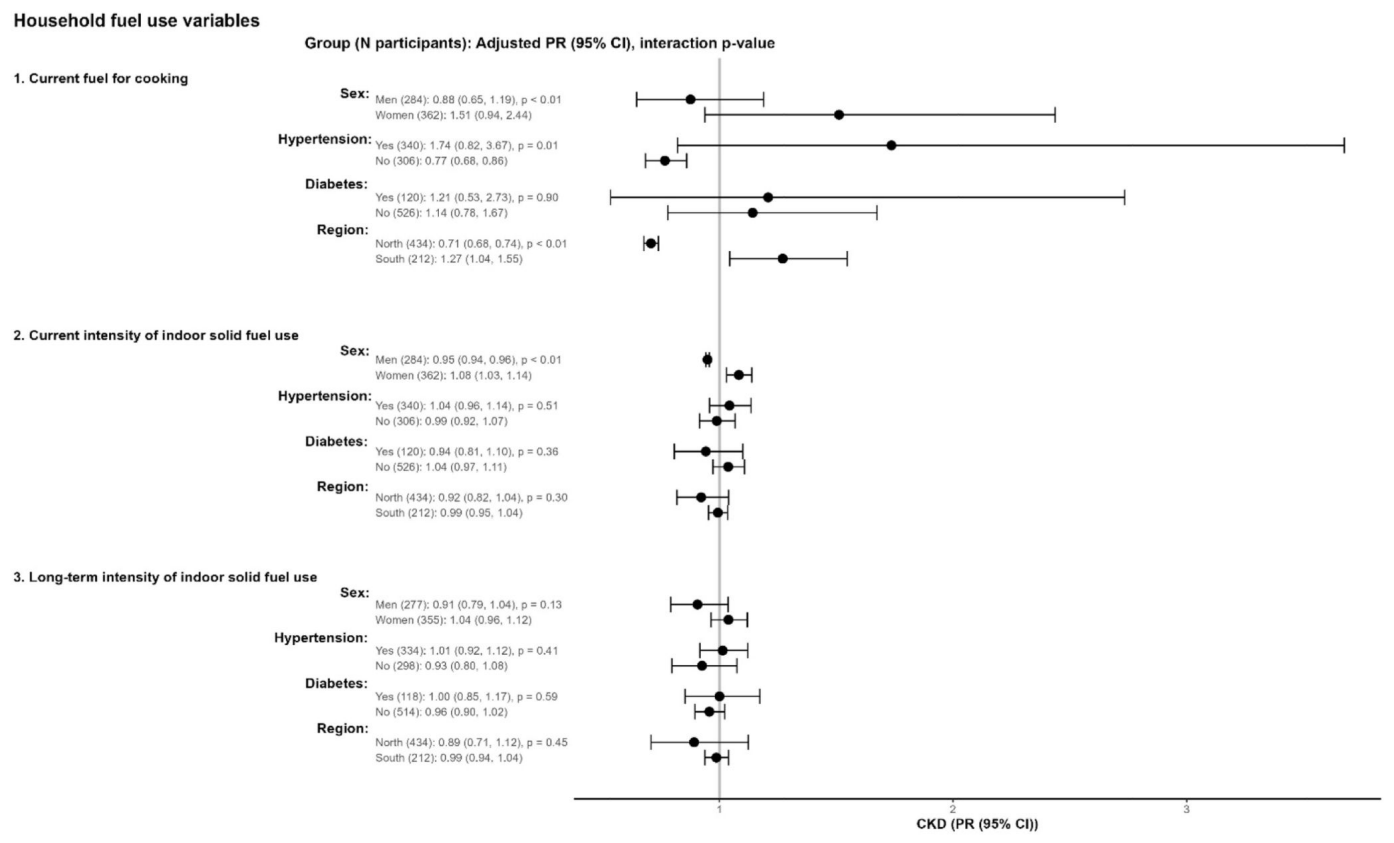
Associations between household solid fuel use and prevalent chronic kidney disease for different subgroups. CI = confidence interval, CKD = chronic kidney disease, PR = prevalence ratio Estimates are for solid fuel exposures compared to reference groups (1 = current exclusive use of clean fuel cookstoves). Number of participants with CKD by cooking fuel: N_clean_ = 14; N_solid_ = 39-Units of exposures for 2 and 3 are per 100 solid fuel stove-use days per year and per 5 solid fuel stove-use years, respectively. Multivariable model adjusted for age, sex, waist circumference, systolic blood pressure, educational attainment, yearly household income, alcohol consumption, history of tobacco smoking, secondhand smoke exposure, and physical activity. All models accounted for provincelevel clustering.

We observed stronger associations between solid fuel use and worse kidney function, as measured by eGFR (Figure 3) and prevalent CKD (Figure 4), in our effect modification analyses, though these associations were not consistent across all fuel use categories. Among females, current solid fuel for cooking and greater intensity of indoor solid fuel use in the past year were associated with higher prevalent CKD. Among participants with hypertension, current use of solid fuel for cooking was associated with lower eGFR and higher prevalent CKD, and greater intensity of indoor solid fuel stove use in the previous year and during the past 20 years were associated with lower eGFR. Among participants with diabetes, current use of solid fuel and greater intensity of long-term solid fuel use were all associated with lower eGFR. In our region-specific analyses, we observed slightly larger associations between solid fuel stove use and prevalent CKD and between intensity of solid fuel stove use and lower eGFR in southern China (Guangxi) compared with participants in the northern sites.

Our results with all participants and for population sub-groups did not appreciably change after limiting the analysis to participants with lower BMI, excluding participants with coronary heart disease or a previous stroke, or additionally adjusting for (1) heating stoves in the cookstoves models, (2) cardiovascular or metabolic conditions including diabetes or hypertension, (3) total cholesterol, HDL cholesterol, LDL cholesterol and triglycerides, or (4) potential mediating variables including diabetes and hypertension (Table S7).

## Discussion

In this cross-sectional study of household fuel use and kidney function in 646 peri-urban and rural Chinese adults, we found some evidence of a modest association between household solid fuel use and worse kidney function in vulnerable population subgroups, including females and participants with hypertension and diabetes. Notable strengths of our study include our comprehensive assessment of household fuel and stove information during the previous 20 years, which overlaps with the preclinical period before the diagnosis of CKD (Brand et al., 2011) and enabled us to more comprehensively measure exposure to household solid fuel use (Carter et al., 2019; Tseng et al., 2022) whereas the few previous studies were limited to primary fuel use (Kim et al., 2016; Xue et al., 2021). We collected blood creatinine to measure eGFR as an objective measure of kidney function and controlled for a comprehensive set of confounders, including measures of socioeconomic position (i.e., educational attainment, household income) and the presence of other health conditions including vascular diseases that were not previously considered in studies on this topic (Liu et al., 2020; Wu et al., 2020; Xue et al., 2021).

Prevalence of CKD (defined as eGFR <60 mL/min/1.73 m^2^) in our participants (8.2%) was slightly higher than in previous studies in rural China (range: 3-5%) that used similar eGFR equations and cut-offs for CKD, and this was also reflected in the higher participant mean eGFR levels in those studies (range: 97.1 to 110.8 mL/min/1.73m^2^) compared with ours (82.4 mL/min/1.73m^2^) (Du et al., 2017; Duan et al., 2019). Underdiagnosis of kidney disease by a physician among our participants could be attributable to a range of factors, including under-reporting by participants or limited access to primary health care and diagnostic tests (Iroegbu et al., 2022).

Prevalence of CKD was higher among our participants in southern China whereas prevalent hypertension and diabetes were lower in the south, which is a geographic trend of observed in other multi-provincial studies of kidney function and prevalent CKD (Chen et al., 2005; Jin et al., 2020). This geographic disparity could be due to a number of factors including differences in health behaviours (e.g., physical activity, dietary and nutrient intake), environmental factors (e.g., air pollution, climate, water quality), and genetics (Zhang et al., 2012). Warmer ambient temperature is generally associated with lower blood pressure (Q. Wang et al., 2017) but could also influence in a higher prevalence of dehydration or higher ambient temperature-related renal function decline in the sub-tropical climate of Guangxi (Borg et al., 2017). Untreated hypertension, which was also higher in southern China participants, in combination with genetics can induce inflammatory and fibrosis pathways leading to glomerular sclerosis and progression to kidney failure (Mennuni et al., 2014). Hypertension-attributed nephropathy has also been linked with increased risk of kidney failure among adults with diabetes (Pirkle and Freedman, 2013). Unknown causes of glomerulonephritis may also increase the risk of CKD in the south (Chen et al., 2005).

We did not find strong or consistent associations of solid fuel cooking on eGFR in the models with all study participants, and only observed more consistent adverse associations among vulnerable subgroups including females, who are more likely to cook household meals than men, and participants with hypertension and diabetes (range of coefficients: -0.6 to -6.6 ml/min/1.73 m^2^ lower eGFR). A study of Chinese adults in a similar age range to ours observed -2.8 ml/min/1.73 m^2^ (95% CI: -5.5, -0.1) lower eGFR (measured using cystatin C) among those primarily cooking with solid fuel compared with those primarily cooking with clean fuel. Similar to our study, they also observed a larger association among participants with hypertension (-3.7 ml/min/1.73 m^2^; 95% CI: - 8.2, 0.8) (Xue et al., 2021), indicating that solid fuel cooking may increase vulnerability to renal function in people with hypertension. Though hypertension has often been considered a confounder in studies of solid fuel–renal function relationships (Xue et al., 2021), whether it is a confounder or mediator, as has been shown for the relationship between solid fuel and diabetes (Bowe et al., 2020), remains to be elucidated. In our study, additionally adjusting for hypertension and diabetes did not attenuate our estimates.

We observed a positive associations between prevalent CKD and both current solid fuel cooking and intensity of indoor solid fuel use in our study, supporting results from a previous cohort study in China which observed an increased risk of kidney disease-related mortality among females reporting to ever have cooked with a coal stove compared with females using clean fuel (hazard ratio: 1.11; 95% CI: 0.66, 1.84) (Kim et al., 2016). Though neither the duration of coal stove use (Kim et al., 2016) nor switching to clean fuel (Xue et al., 2021) were associated with changes in kidney disease-related mortality or CKD risk Chinese adults in previous studies. Further supporting our findings, however, is a recent meta-analysis of outdoor air pollution and renal function that found 4.11 mL/min/1.73 m^2^ lower eGFR (95% CI: –12.64, 4.42) and a 10% higher CKD risk (95% CI: 1.00, 1.21) per 10 μg/m^3^ increase in outdoor PM_2.5_, with most studies included in the meta-analysis considering hypertension and diabetes as confounders (Wu et al., 2020).

The larger associations between solid fuel use and CKD observed among females could be explained by behavioral or biological factors. Women tend to spend more time cooking and working indoors in rural China, which may increase their exposure to pollution from solid fuel cookstoves (Bowe et al., 2018) and thus increase their risk of CKD compared with men (Bikbov et al., 2020; Chen et al., 2005). Experimental studies indicate that females also have slightly greater airway reactivity and deposition of particulates in the lung than males, which could increase their susceptibility to air pollution (Sturm, 2016). Generally we did not observe large or consistent differences in the associations by age, which may be because our study participants were generally older. This contradicts previous studies showing that older adults had greater systemic inflammation-related renal decline (Costello-White et al., 2015), but aligns with results from a large cross-sectional analysis where the associations between cooking and heating stove-specific exposures and eGFR were similar for older and younger Chinese adults (Xue et al., 2021).

The larger associations between current fuel use and worse kidney function observed among participants in the southern site of Guangxi in our study could be related to the overall larger burden of CKD and wider range of eGFR among participants in the south. This finding also raises the question of whether fuel type may differentially affect health. Guangxi residents primarily cook with wood and other biomass fuel while Beijing and Shanxi participants heavily rely on coal and biomass stoves. Coal and biomass combustion emit different levels and chemical compositions of pollutants (Du et al., 2018; Shen et al., 2021). However, we were unable to distinguish the renal impacts of coal versus biomass because mixed use of both fuel types was common among participants in northern China, with very few reporting to use only coal (Lai et al., 2019).

The exact biological mechanisms through which household air pollution affects kidney function are unclear, though a direct causal effect is plausible (Afsar et al., 2019). Incomplete combustion of solid fuels may directly release toxicants that can enter the blood circulation through the lungs, causing glomerulosclerosis and renal tubular damage (Yang Ya-Ru et al., 2017). Toxicants from household air pollution may also stimulate pro-inflammatory pathways in the lung or cause mitochondrial damage and autoimmune responses that ultimately induces kidney damage (Xue et al., 2021). Exposure to air pollution (both ambient and household) is also known to elevate blood pressure, which is a risk factor for renal function decline (Afsar et al., 2019); this is supported by our observations of lower eGFR and higher CKD among hypertensive versus normotensive participants.

China accounts for a fifth of the global burden of CKD and, in the absence of intervention, its CKD burden is expected to increase as its population continues to urbanize and age (Bikbov et al., 2020; Zhang et al., 2012). Population-level interventions that can effectively mitigate renal function decline are urgently needed. Our study indicates that the provision of clean household energy may be one solution, especially for vulnerable populations. China is well-positioned to implement large-scale clean household energy programs through established investments in clean energy infrastructure and technology (Jin et al., 2006) given the strong historical precedent of having already successfully transitioned hundreds of millions of Chinese households to clean energy over the past two decades (Stoner et al., 2021).

Our study has several limitations to consider for future studies. Although many potential confounders were statistically controlled for in the analyses, we cannot eliminate the possibility of bias due to residual confounding in our observational study. Specifically, we did not measure and thus control for indoor temperature, outdoor air pollution, or dietary factors, such as, processed food intake, that may protect or harm renal health. Though unlikely, these factors could conceivably confound the relationship between current fuel use and kidney function, but would not influence long-term fuel use and kidney function. Second, our single assessment of kidney function precluded us from evaluating household fuel use-related differences in the trajectories of kidney decline and reverse causality is also possible, though small differences in eGFR are unlikely to affect stove use behaviours, especially those over the longer term. We may have underestimated the prevalence of CKD and the levels of eGFR in our study because we were unable to measure albuminuria, an indicator of kidney damage (J. Wang et al., 2017), and cystatin C, which may improve the accuracy of eGFR equations since serum creatinine levels are affected by age, sex, and protein intake (Chi et al., 2017; Chowdhury et al., 2019; Xue et al., 2021). Though this measurement error in our outcomes of eGFR and CKD are unlikely related to exposure to solid fuel. We also had a relatively small number of participants with prevalent CKD in our study, especially within fuel use categories (N_clean_=14; N_solid_ = 39). This reduces our statistical power and lowers our chance of detecting an effect but also increases the likelihood that any observed associations are due to chance. We did observe some consistency in the sub-group results for prevalent CKD and continuous eGFR, but the small number of CKD outcomes in subgroups is nonetheless a limitation that warrants a cautious interpretation of the prevalent CKD analyses. Additionally, samples from different provinces were analyzed in different laboratories but with similar standard procedures, potentially contributing to systematic differences in the creatinine levels between provinces. To minimize this and other potential bias related to province, we conducted GEE models with province-level clusters and separately analyzed the data by geographic region when feasible.

Some degree of exposure misclassification is expected in our self-reported stove-use variables, which were designed to estimate longer-term exposure to household air pollution. We were able to externally verify a sub-sample of self-reported data based on household visits and village records of electricity and gas use (Carter et al., 2019), but could not externally verify all historical stoves use reports. Most misclassification in stove use is likely non-differential and would usually result in a bias toward the null, but it is possible that people with poorer health may less accurately report stove use due to cognitive or mental health burden, which could lead to bias in either direction. We also assumed that intensity of use for a given stove remained constant over 5-year periods, which likely contributed to exposure misclassification due to changes in use or intensity of use during that period, though this error is also likely non-differential and would likely bias our results toward the null.

## Conclusions

Our study contributes to the very limited understanding of the association of renal function with household solid fuel use, a pervasive environmental exposure that impacts nearly four billion people globally. We did not observe strong or consistent associations between household solid fuel use and renal function among all study participants in this cross-sectional study. Household solid fuel use and intensity of use were associated with modestly lower kidney function only in females and in participants with hypertension and diabetes, though many confidence intervals included the null.

## Supplementary Material

Supplementary Material

## Figures and Tables

**Table 1:** Characteristics of the study participants at the time of first survey, by cooking fuel use (mean (SD) for continuous variables or n (%) for categorical variables).

	Exclusive use of clean fuel (n=211)	Any use of solid fuel (n=435)	All (n=646)
Age (years)	61.6 (9.1)	63.0 (8.2)	62.6 (8.5)
Sex (% female)	114 (54.0%)	248 (57.0%)	362 (56.0%)
Province			
Beijing	59 (28.0%)	159 (36.6%)	218 (33.7%)
Guangxi	65 (30.8%)	147 (33.8%)	212 (32.8%)
Shanxi	87 (41.2%)	129 (29.7%)	216 (33.4%)
Yearly household income (RMB)			
<20,000	79 (37.4%)	201 (46.2%)	280 (43.3%)
≥20,000	132 (62.6%)	234 (53.8%)	366 (56.7%)
Highest educational attainment			
No formal education	31 (14.7%)	71 (16.3%)	102 (15.8%)
Primary school	84 (39.8%)	183 (42.1%)	267 (41.3%)
High school or college	96 (45.5%)	181 (41.6%)	277 (42.9%)
History of tobacco smoking			
Never	122 (57.8%)	273 (62.8%)	395 (61.1%)
Former	35 (16.6%)	72 (16.6%)	107 (16.6%)
Current	54 (25.6%)	90 (20.7%)	144 (22.3%)
Secondhand smoke exposure			
Never	121 (57.3%)	238 (54.7%)	359 (55.6%)
Former	39 (18.5%)	75 (17.2%)	114 (17.6%)
Current	51 (24.2%)	122 (28.0%)	173 (26.8%)
Alcohol consumption (past year)			
Never	131 (62.1%)	261 (60.0%)	392 (60.7%)
Occasional (<1 drink per week)	39 (18.5%)	100 (23.0%)	139 (21.5%)
Regular (≥1 drink per week)	41 (19.4%)	74 (17.0%)	115 (17.8%)
Physical activity (frequency in past 3 months)			
None	52 (24.6%)	66 (15.2%)	118 (18.3%)
≤2 times per week	39 (18.5%)	97 (22.3%)	136 (21.1%)
≥3 times per week	120 (56.9%)	272 (62.5%)	392 (60.7%)
Hypertension (% yes)	110 (52.1%)	230 (52.9%)	340 (52.6%)
Current use of anti-hypertensive medication (% yes)	81 (38.4%)	157 (36.1%)	238 (36.8%)
History of coronary heart disease or stroke (% yes)	25 (11.8%)	52 (12.0%)	77 (11.9%)
Physician-diagnosed diabetes or fasting plasma glucose ≥7.0 mmol/L (% yes)	41 (19.4%)	79 (18.2%)	120 (18.6%)
Waist circumference (cm)	87.9 (9.5)	86.9 (9.9)	87.2 (9.8)
Body mass index (kg/m^2^)	25.6 (3.6)	25.2 (3.9)	25.3 (3.8)
Systolic blood pressure (mmHg)	130.4 (17.5)	133.8 (18.2)	132.7 (18.0)
Triglycerides (mmol/L)	1.7 (2.0)	1.5 (1.4)	1.5 (1.6)
Total cholesterol (mmol/L)	5.1 (1.1)	5.0 (1.1)	5.0 (1.1)
LDL cholesterol (mmol/L)	3.0 (1.0)	3.0 (0.9)	3.0 (0.9)
HDL cholesterol (mmol/L)	1.3 (0.3)	1.3 (0.3)	1.3 (0.3)
Physician-diagnosed kidney disease (% yes)	3 (1.4%)	19 (4.4%)	22 (3.4%)
eGFR (ml/min/1.73 m2)	83.5 (15.0)	81.9 (15.3)	82.4 (15.2)
Chronic kidney disease (eGFR <60 ml/min/1.73 m2) (% yes)	14 (6.6%)	39 (9.0%)	53 (8.2%)

RMB = Chinese Yuan, Physical activity = exercise and farm-based physical activity domains, eGFR = estimated glomerular filtration rate, hypertension = current use of anti-hypertensive medication, systolic (≥140 mmHg) or diastolic (≥90 mmHg) blood pressure, cardiovascular disease = a history of coronary heart disease or stroke, LDL = low-density lipoproteins, SD = standard deviation.

**Table 2:** Descriptive statistics for kidney function and exposure to household fuel use among older Chinese adults [mean (SD), median (min, max), or n (%)], by sex and study site.

	Beijing		Guangxi		Shanxi	
	Males (n=87)	Females (n=131)	Males (n=99)	Females (n=113)	Males (n=98)	Females (n=118)
**Kidney function**						
eGFR (ml/min/1.73 m^2^)	84.3 (12.4)	85.5 (12.4)	75.1 (18.5)	77.0 (18.2)	86.8 (12.5)	85.4 (12.3)
Chronic kidney disease based on eGFR <60 ml/min/1.73 m^2^	3 (3.4%)	6 (4.6%)	19 (19.2%)	19 (16.8%)	1 (1.0%)	5 (4.2%)
**Household fuel use exposures**						
Current cookstove use						
Any current use of solid fuel	63 (72.4%)	96 (73.3%)	66 (66.7%)	81 (71.7%)	58 (59.2%)	71 (60.2%)
Exclusive use of clean fuel	24 (27.6%)	35 (26.7%)	33 (33.3%)	32 (28.3%)	40 (40.8%)	47 (39.8%)
Current heating stove use	
Any current use of solid fuel	81 (93.1%)	124 (94.7%)	-	-	74 (75.5%)	92 (78.0%)
Exclusive use of clean fuel	6 (6.9%)	6 (4.6%)	-	-	19 (19.4%)	18 (15.3%)
No heating stoves	0 (0%)	1 (0.8%)	-	-	5 (5.1%)	8 (6.8%)
Current (past year) intensity of indoor solid fuel use (solid fuel stove-use days per year)	223.2 (176.2)	198.4 (189.7)	253.9 (217.5)	283.4 (208.0)	262.8 (231.9)	295.2 (225.6)
Long-term (20 y) intensity of indoor solid fuel use (solid fuel stove-use years)	13.3 [1.5, 35.0]	15.0 [0.8, 52.5]	15.0 [0.0, 35.0]	15.0 [0.0, 35.0]	20.0 [2.5, 29.1]	20.0 [0.8, 33.3]

eGFR = estimated glomerular filtration rate, SD = standard deviation.
