# Supplementary material for: Cross-sectional study of household solid fuel use and renal function in older adults in China
Source: Environ Res. Author manuscript; Available in PMC 2023 Oct 27. (PMC7615253; doi:10.1016/j.envres.2022.115117)
Supplement: Supplementary Material [file EMS188998-supplement-Supplementary_Material.docx]

Table S1. Household fuel use and intensity of use exposure variables and their descriptions. Detailed descriptions of the development of these exposure variables are provided in(Kanagasabai et al., 2022; Tseng et al., 2022).

| **Variable** | **Description** |
| --- | --- |
| 1. Current fuel for cooking | Any use of solid fuel cookstoves (n=435)  Exclusive use of clean fuel cookstoves **^a^** (n=211; reference) |
| 1. Current fuel for heating (northern China) | Any use of solid fuel heating stoves (n=371)  Exclusive clean fuel heating stoves (n=49; reference)  No heating stoves (n=14)^b^ |
| 1. Current intensity of indoor solid fuel stove use | Number of indoor solid fuel stove-use days in the past year, continuous |
| 1. Long-term intensity of indoor solid fuel stove use | Number of indoor solid fuel stove-use years in the past 20 years, continuous |
| ^a^Clean fuel stoves included those powered by gas or electricity and solid fuel stoves included those powered by coal, wood, crop residues, or other forms of biomass. ^b^This group was excluded from the analysis given the small sample size. | |

Table S2. Chinese population specific Chronic Kidney Disease Epidemiology Collaborationequations for the estimation of glomerular filtration rate (ml/min/1.73 m^2^).

| **Sex** | **Condition** | **Formula** |
| --- | --- | --- |
| Female  Male | CR≤61.88 µmol/L  CR>61.88 µmol/L  CR≤79.56 µmol/L  CR>79.56 µmol/L | 141 × (CR/61.88)^-0.329^ × 0.993^age^ × 1.018  141 × (CR/61.88)^-1.209^ × 0.993^age^ × 1.018  141 × (CR/79.56)^-0.411^ × 0.993^age^  141 × (CR/79.56)^-1.209^ × 0.993^age^ |
| CR = serum creatinine. | | |

**Table S3**. Select summary statistics for participants with missing household income information versus those with complete household income data (mean (standard deviation) for continuous variables or n (%) for categorical variables).

| **Variable** | **Beijing** | | **Shanxi** | | **Guangxi** | |
| --- | --- | --- | --- | --- | --- | --- |
|  | **Missing**  **N=13** | **Complete**  **N=205** | **Missing**  **N=41** | **Complete**  **N=175** | **Missing**  **N=15** | **Complete**  **N=197** |
| eGFR (ml/min/1.73 m^2^) | 85.9 (12.9) | 85.0 (12.4) | 83.5 (14.0) | 86.6 (11.9) | 75.1 (16.4) | 76.2 (18.5) |
| Age (years) | 66.3 (7.4) | 62.6 (7.5) | 63.8 (8.0) | 61.5 (8.5) | 62.4 (10.3) | 62.9 (9.6) |
| Sex (% female) | 10 (77%) | 121 (59%) | 22 (54%) | 96 (55%) | 8 (53%) | 105 (53%) |
| Smoking status |  |  |  |  |  |  |
| Current | 2 (16%) | 42 (21%) | 11 (27%) | 51 (29%) | 4 (27%) | 34 (17%) |
| Never/Former | 11 (84%) | 163 (80%) | 30 (72%) | 124 (71%) | 11 (73%) | 163 (82%) |

**Table S4.** Distributions of covariates for observed and imputed data.

|  | Observations | Imputed estimates for missing observations |
| --- | --- | --- |
| Annual income  <20,000 RMB (%)  ≥20,000 RMB (%) | 42%  58% | 40%  60% |
| Waist circumference (cm)  Mean (SD.) | 87.2 (9.6) | 87.2 (9.8) |
| Body mass index (kg/m^2^)  Mean (SD) | 25.3 (3.8) | 25.3 (3.8) |

**Table S5**. *STROBE* checklist for the present study.

|  | **Item No** | **Recommendation** |
| --- | --- | --- |
| **Title and abstract** | 1 | (*a*) Indicate the study’s design with a commonly used term in the title or the abstract  *The title of the study is described as cross-sectional analysis of household solid fuel use and renal decline.* |
|  |  | (*b*) Provide in the abstract an informative and balanced summary of what was done and what was found  *The abstract describes the methods and finding.* |
| **Introduction** | | |
| Background/rationale | 2 | Explain the scientific background and rationale for the investigation being reported  *The background and rationale are described in the Introduction, paragraphs 1 to 3.* |
| Objectives | 3 | State specific objectives, including any prespecified hypotheses  *The specific aims of the study are stated in the Introduction, paragraph 4.* |
| **Methods** | | |
| Study design | 4 | Present key elements of study design early in the paper  *The study design is discussed in paragraph 4 of the Introduction and paragraphs*  *2 of the Methods section.* |
| Setting | 5 | Describe the setting, locations, and relevant dates, including periods of recruitment, exposure, follow-up, and data collection  *The institutional setting is described in paragraphs 2 of the Methods section;*  *Study locations are described in paragraph 1 of the Methods section; and study*  *timing is discussed in paragraph 3 of the Methods section* |
| Participants | 6 | (*a*) Give the eligibility criteria, and the sources and methods of selection of participants  *Selection of the sample is discussed in paragraph 2 of the Methods section.* |
| Variables | 7 | Clearly define all outcomes, exposures, predictors, potential confounders, and effect modifiers. Give diagnostic criteria, if applicable  *Outcomes are described in paragraph 5 of Methods section. Exposures are described in paragraph 4 of Methods section, and covariates considered in the study are described in paragraphs 6-7 of Methods section.* |
| Data sources/ measurement | 8* | For each variable of interest, give sources of data and details of methods of assessment (measurement). Describe comparability of assessment methods if there is more than one group  *Data measurements are described in paragraphs 3-7 of Methods section, under appropriate subsections.* |
| Bias | 9 | Describe any efforts to address potential sources of bias  *Potential sources of bias are described in paragraphs 9-10 of Discussion section.* |
| Study size | 10 | Explain how the study size was arrived at  *Sample determination is discussed in paragraph 2 of the Methods section.* |
| Quantitative variables | 11 | Explain how quantitative variables were handled in the analyses. If applicable, describe which groupings were chosen and why  *Use of variables is discussed in the Statistical Analysis subsection. Additional details of variable selection are provided in paragraphs 4-7 of Methods section.* |
| Statistical methods | 12 | (*a*) Describe all statistical methods, including those used to control for confounding  *Statistical methods are discussed in the Statistical Analysis subsection* |
|  |  | (*b*) Describe any methods used to examine subgroups and interactions  *Described in paragraph 1 of the Statistical Analysis subsection. All models accounted for province level clusters.* |
|  |  | (*c*) Explain how missing data were addressed  *Described in paragraph 1 of the Statistical Analysis subsection.* |
|  |  | (*d*) If applicable, describe analytical methods taking account of sampling strategy  *None.* |
|  |  | (*e*) Describe any sensitivity analyses  *Described in paragraph 2 of the Statistical Analysis subsection.* |
| **Results** | | |
| Participants | 13* | (a) Report numbers of individuals at each stage of study—eg numbers potentially eligible, examined for eligibility, confirmed eligible, included in the study, completing follow-up, and analysed  *Described in paragraph 1 of Results section, and paragraph 2 of Methods section.* |
|  |  | (b) Give reasons for non-participation at each stage  *Described in paragraph 2 of Methods section.* |
|  |  | (c) Consider use of a flow diagram  *Full details about the participants are provided in the protocol paper (Yan et al., 2019), and in this paper in paragraph2 of Methods section and paragraph 1 of Results section.* |
| Descriptive data | 14* | (a) Give characteristics of study participants (eg demographic, clinical, social) and information on exposures and potential confounders  *Provided in Tables 1 and 2, and in paragraphs 1-4 of Results section.* |
|  |  | (b) Indicate number of participants with missing data for each variable of interest  *Provided in Table S1 of Kanagasabai et al. 2022.* |
| Outcome data | 15* | Report numbers of outcome events or summary measures  *Provided in Table 2 and described in paragraph 4 of Results section.* |
| .Main results | 16 | (*a*) Give unadjusted estimates and, if applicable, confounder-adjusted estimates and their precision (eg, 95% confidence interval). Make clear which confounders were adjusted for and why they were included.  *Provided in Figures 1 and 2, and described in paragraph 5 of Results section.* |
|  |  | (*b*) Report category boundaries when continuous variables were categorized  *Provided throughout the manuscript for CKD as eGFR <60 ml/min/1.73 m2.* |
|  |  | (*c*) If relevant, consider translating estimates of relative risk into absolute risk for a meaningful time period  *Not applicable.* |
| Other analyses | 17 | Report other analyses done—eg analyses of subgroups and interactions, and sensitivity analyses  *Provided in Figures 3 and 4, Tables S4, and described in paragraphs 6 and 7 of the Results section.* |
| **Discussion** | | |
| Key results | 18 | Summarise key results with reference to study objectives  *Provided in paragraph 1 of Discussion section.* |
| Limitations | 19 | Discuss limitations of the study, taking into account sources of potential bias or imprecision. Discuss both direction and magnitude of any potential bias  *Provided in paragraphs 9-10 of Discussion section.* |
| Interpretation | 20 | Give a cautious overall interpretation of results considering objectives, limitations, multiplicity of analyses, results from similar studies, and other relevant evidence  *Final paragraph of Discussion section (Conclusion subsection).* |
| Generalisability | 21 | Discuss the generalisability (external validity) of the study results  *Provided at the end of paragraph 9 in Discussion section.* |
| **Other information** | | |
| Funding | 22 | Give the source of funding and the role of the funders for the present study and, if applicable, for the original study on which the present article is based  *Provided in Acknowledgements section.* |

*Give information separately for exposed and unexposed groups.

**Note:** An Explanation and Elaboration article discusses each checklist item and gives methodological background and published examples of transparent reporting. The STROBE checklist is best used in conjunction with this article (freely available on the Web sites of PLoS Medicine at http://www.plosmedicine.org/, Annals of Internal Medicine at http://www.annals.org/, and Epidemiology at http://www.epidem.com/). Information on the STROBE Initiative is available at www.strobe-statement.org.

Table S6. Characteristics of the study participants by province and sex (mean (SD), median [min, max], or n (%)).

|  | **Beijing** | | **Guangxi** | | **Shanxi** | |
| --- | --- | --- | --- | --- | --- | --- |
|  | **Male** | **Female** | **Male** | **Female** | **Male** | **Female** |
|  | **(n=87)** | **(n=131)** | **(n=99)** | **(n=113)** | **(n=98)** | **(n=118)** |
| Age (years) | 64.0 (7.4) | 62.0 (7.5) | 62.4 (9.7) | 63.3 (9.5) | 62.4 (8.9) | 61.5 (8.0) |
| Yearly household income (CYN) |  |  |  |  |  |  |
| <20,000 | 52 (59.8%) | 57 (43.5%) | 12 (12.1%) | 12 (10.6%) | 71 (72.4%) | 76 (64.4%) |
| ≥20,000 | 35 (40.2%) | 74 (56.5%) | 87 (87.9%) | 101 (89.4%) | 27 (27.6%) | 42 (35.6%) |
| Highest educational attainment |  |  |  |  |  |  |
| No formal education | 8 (9.2%) | 46 (35.1%) | 4 (4.0%) | 19 (16.8%) | 4 (4.1%) | 21 (17.8%) |
| Primary school | 38 (43.7%) | 32 (24.4%) | 40 (40.4%) | 51 (45.1%) | 40 (40.8%) | 66 (55.9%) |
| Early high school, college, or above | 41 (47.1%) | 53 (40.5%) | 55 (55.6%) | 43 (38.1%) | 54 (55.1%) | 31 (26.3%) |
| History of tobacco smoking |  |  |  |  |  |  |
| Never | 15 (17.2%) | 131 (100%) | 23 (23.2%) | 113 (100%) | 6 (6.1%) | 107 (90.7%) |
| Former | 28 (32.2%) | 0 (0%) | 38 (38.4%) | 0 (0%) | 36 (36.7%) | 5 (4.2%) |
| Current | 44 (50.6%) | 0 (0%) | 38 (38.4%) | 0 (0%) | 56 (57.1%) | 6 (5.1%) |
| Secondhand smoke exposure |  |  |  |  |  |  |
| Never | 84 (96.6%) | 26 (19.8%) | 89 (89.9%) | 28 (24.8%) | 97 (99.0%) | 35 (29.7%) |
| Former | 0 (0%) | 40 (30.5%) | 2 (2.0%) | 40 (35.4%) | 0 (0%) | 32 (27.1%) |
| Current | 3 (3.4%) | 65 (49.6%) | 8 (8.1%) | 45 (39.8%) | 1 (1.0%) | 51 (43.2%) |
| Alcohol consumption (past year) |  |  |  |  |  |  |
| Never | 20 (23.0%) | 115 (87.8%) | 31 (31.3%) | 87 (77.0%) | 33 (33.7%) | 106 (89.8%) |
| Occasional (<1 drink per week) | 24 (27.6%) | 14 (10.7%) | 29 (29.3%) | 23 (20.4%) | 39 (39.8%) | 10 (8.5%) |
| Regular (≥1 drink per week) | 43 (49.4%) | 2 (1.5%) | 39 (39.4%) | 3 (2.7%) | 26 (26.5%) | 2 (1.7%) |
| Physical activity (frequency in past 3 months) |  |  |  |  |  |  |
| None | 14 (16.1%) | 23 (17.6%) | 20 (20.2%) | 12 (10.6%) | 19 (19.4%) | 30 (25.4%) |
| ≤2 times per week | 9 (10.3%) | 20 (15.3%) | 20 (20.2%) | 15 (13.3%) | 32 (32.7%) | 40 (33.9%) |
| ≥3 times per week | 64 (73.6%) | 88 (67.2%) | 59 (59.6%) | 86 (76.1%) | 47 (48.0%) | 48 (40.7%) |
| Hypertension (% yes) | 57 (65.5%) | 88 (67.2%) | 41 (41.4%) | 38 (33.6%) | 44 (44.9%) | 72 (61.0%) |
| Current use of anti-hypertensive medication (% yes) | 38 (43.7%) | 68 (51.9%) | 8 (8.1%) | 15 (13.3%) | 41 (41.8%) | 68 (57.6%) |
| Self-reported history of coronary heart disease or stroke (% yes) | 14 (16.1%) | 16 (12.2%) | 8 (8.1%) | 5 (4.4%) | 16 (16.3%) | 18 (15.3%) |
| Physician-diagnosed diabetes or fasting plasma glucose ≥7.0 mmol/L (% yes) | 20 (23.0%) | 28 (21.4%) | 13 (13.1%) | 10 (8.8%) | 17 (17.3%) | 32 (27.1%) |
| Waist circumference (cm) | 90.5 (8.2) | 89.2 (9.4) | 83.5 (9.5) | 82.3 (8.7) | 89.5 (11.3) | 88.7 (8. 6) |
| Body mass index (kg/m^2^) | 25.8 (3.1) | 26.9 (3.9) | 23.3 (3.9) | 23.5 (3.5) | 25.2 (3.8) | 26.6 (3.3) |
| Systolic blood pressure (mmHg) | 139.5 (16.4) | 138.2 (17.0) | 135.3 (17.7) | 132.0 (18.9) | 123.2 (14.6) | 127.8 (17.8) |
| Physician-diagnosed kidney disease (% yes) | 5 (5.7%) | 3 (2.3%) | 8 (8.1%) | 4 (3.5%) | 2 (2.0%) | 0 (0%) |

Table S7. Results from the main analysis (bold text) and fromsensitivity analyses for models of exposure to household solid fuel use and a) estimated glomerular filtration rate and b) prevalence ratio of chronic kidney disease.

| **Description of models** | **N** | **Any use of solid fuel cookstoves^1^** | **Any use of solid fuel heating stoves^2^** | **Current intensity of indoor solid fuel use**  **(per 100 sold fuel stove-use daysper year)** | **Long-term intensity of indoor solid fuel use**  **(per 5 solid fuel stove-use years)** |  |  |
| --- | --- | --- | --- | --- | --- | --- | --- |
|  |  | **a) Estimated glomerular filtration rate, ml/min/1.73 m^2^ (95% CI)** | | | | |  |
| **All participants^3^** | 646 | 0.17 (-0.30, 0.64) | -0.02 (-2.57, 2.53) | -0.08 (-0.21, 0.06) | -0.25 (-0.71, 0.21) |  |  |
| Additionally adjusted for diabetes | 646 | 0.19 (-0.14, 0.52) | -0.01 (-2.35, 2.34) | -0.08 (-0.21, 0.05) | -0.26 (-0.72, 0.19) |  |  |
| Additionally adjusted for hypertension | 646 | 0.29 (-0.20, 0.78) | -0.01 (-2.71, 2.70) | -0.05 (-0.21, 0.12) | -0.25 (-0.69, 0.19) |  |  |
| Additionally adjusted for clinical variables^4^ | 646 | 0.21 (-0.36, 0.78) | -0.07 (-2.90, 2.76) | -0.07 (-0.11, -0.02) | -0.35 (-0.74, 0.03) |  |  |
| Excluding participants with coronary heart disease or previous stroke | 569 | 0.23 (-0.27, 0.73) | 1.32 (-0.45, 3.08) | 0.06 (-0.04, 0.16) | -0.20 (-0.70, 0.30) |  |  |
| Additionally adjusted for heating fuel | 434 | 0.22 (0.09, 0.35) | - | - | - |  |  |
| Participants with BMI ≥28 kg/m^2^ | 159 | 0.31 (-3.64, 4.26) | -0.26 (-2.45, 1.93) | -0.45 (-1.18, 0.29) | -0.75 (-0.91, -0.58) |  |  |
| **Participants with hypertension^5^** | 340 | -1.52 (-3.55, 0.50) | -0.42 (-5.16, 4.32) | -0.61 (-0.97, -0.25) | -0.91 (-1.36. -0.47) |  |  |
| Additionally adjusted for diabetes | 340 | -1.51 (-3.59, 0.56) | -0.37 (-5.43, 4.68) | -0.61 (-0.99, -0.22) | -0.90 (-1.32, -0.49) |  |  |
| **Participants with diabetes^5^** | 120 | -6.79 (-9.50, -4.09) | -4.59 (-4.71, -4.47) | -0.69 (-1.43, 0.06) | -0.65 (-1.12, -0.17) |  |  |
| Additionally adjusted for hypertension | 120 | -6.72 (-9.34, -4.09) | -4.73 (-4.80, -4.66) | -0.68 (-1.45, 0.09) | -0.65 (-1.16, -0.14) |  |  |
|  |  | **b) Prevalence ratio of chronic kidney disease (95% CI)** | | | | |  |
| **All participants^3^** | 646 | 1.19 (0.86, 1.64) | - | 1.02 (1.00, 1.04) | 0.98 (0.94, 1.02) |  |  |
| Additionally adjusted for diabetes | 646 | 1.16 (0.83, 1.62) |  | 1.01 (0.97, 1.05) | 0.97 (0.91, 1.03 |  |  |
| Additionally adjusted for hypertension | 646 | 1.17 (0.88, 1.57) | - | 1.02 (1.00, 1.03) | 0.98 (0.94, 1.02 |  |  |
| Additionally adjusted for clinical variables^4^ | 646 | 1.13 (0.78, 1.64) | - | 1.02 (0.95, 1.08) | 0.98 (0.87, 1.09) |  |  |
| Excluding participants with coronary heart disease or previous stroke | 569 | 1.62 (1.46, 1.79) | - | 1.02 (1.00, 1.05) | 0.98 (0.92, 1.03) |  |  |
| Participants with BMI ≥28 kg/m^2^ | 159 | 1.14 (0.36, 3.62) | - | 1.10 (0.90, 1.36) | 0.90 (0.76, 1.08) |  |  |
| **Participants with hypertension^5^** | 340 | 1.59 (0.92, 2.72) | - | 1.08 (0.99, 1.18) | 1.07 (0.94, 1.22) |  |  |
| Additionally adjusted for diabetes | 340 | 1.52 (0.87, 2.64) | - | 1.07 (0.95, 1.21) | 1.07 (0.91, 1.24) |  |  |
| **Participants with diabetes^5^** | 120 | 1.36 (0.51, 3.61) | - | 0.89 (0.79, 1.01) | 0.90 (0.74, 1.10) |  |  |
| Additionally adjusted for hypertension | 120 | 1.40 (0.50, 3.93) | - | 0.90 (0.78, 1.03) | 0.91 (0.68, 1.21) |  |  |
| BMI, body mass index; CI = confidence interval  ^1^Reference group= exclusive use of clean fuel cookstoves.  ^2^Reference group = exclusive use of clean fuel heating stoves.  ^3^Multivariable models adjusted for age, sex, waist circumference, systolic blood pressure, yearly household income, educational attainment, alcohol consumption, history of tobacco smoking, secondhand smoke exposure, and physical activity.  ^4^Clinical variables = self-reported history of coronary heart disease, myocardial infarction, stroke, or transient ischemic attack; total cholesterol; HDL cholesterol; LDL cholesterol; and triglycerides.  ^5^Modelled with interaction terms between the comorbid conditions of hypertension or diabetes and exposure to solid fuel. | | | | | | | |

**Figure S1.** Directed acyclic graph (DAG) to illustrate regression modelling of fuel use and renal function.
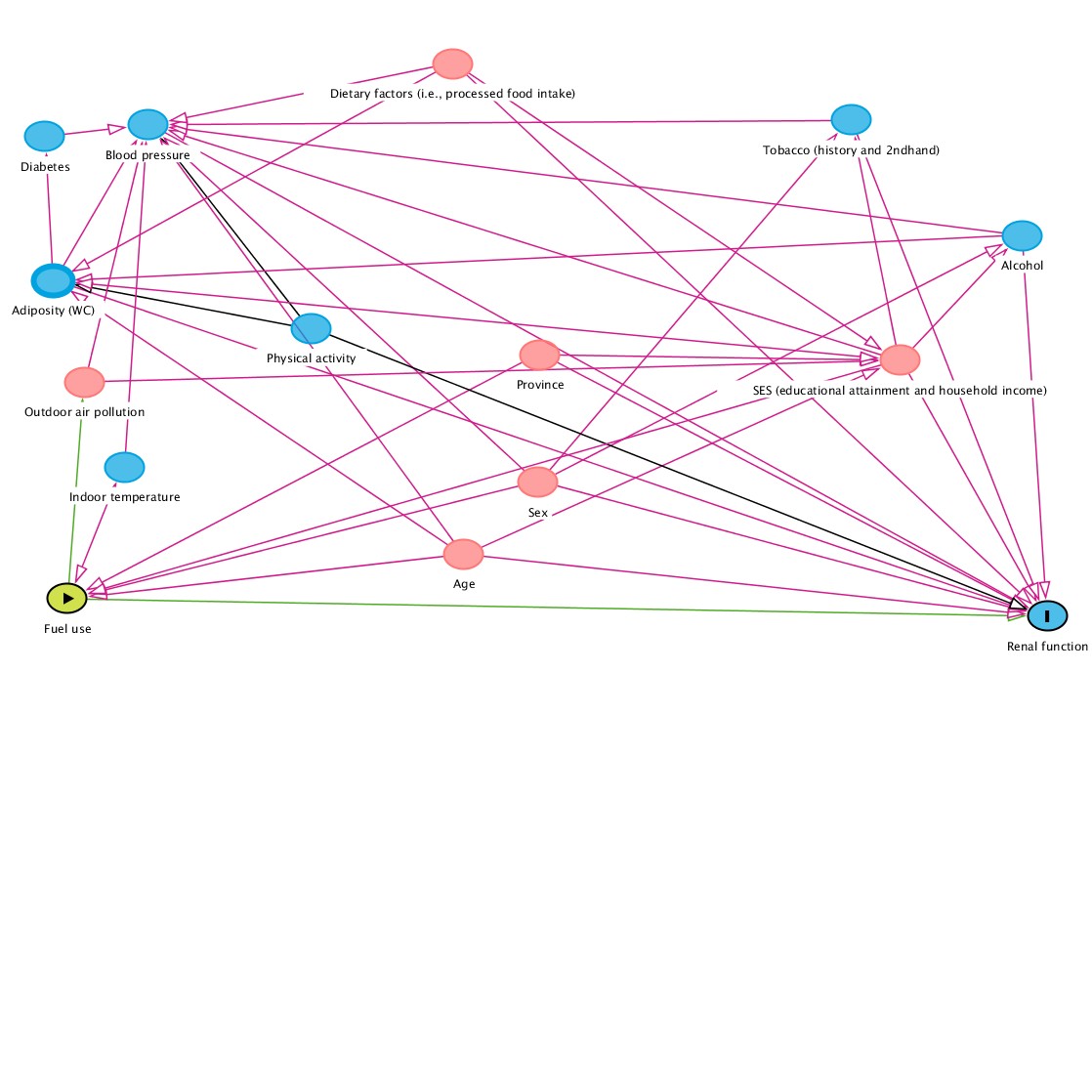


Abbreviation: WC; waist circumference, SES; socioeconomic status measured by education and households income; 2ndhand; secondhand

Directed acyclic graphs (DAGitty: <http://www.dagitty.net/>) to guide and illustrate regression modelling showing potential confounders for fuel use exposures and renal function. Pink lines indicate potential confounders, and green lines indicate the main association, as well as paths through factors along the causal pathway. Data on smoking exposure and environmental smoking exposure confounders were combined in a hybrid variable for adjustment in the analysis. Blood pressure could be a confounder or on the causal pathway.
